# The Multiple Determinants of Maternal Parenting Stress 12 Months After Birth: The Contribution of Antenatal Attachment Style, Adverse Childhood Experiences, and Infant Temperament

**DOI:** 10.3389/fpsyg.2018.01987

**Published:** 2018-10-23

**Authors:** Vibeke Moe, Tilmann von Soest, Eivor Fredriksen, Kåre S. Olafsen, Lars Smith

**Affiliations:** ^1^Department of Psychology, University of Oslo, Oslo, Norway; ^2^The Regional Centre for Child and Adolescent Mental Health, Eastern and Southern Norway, Oslo, Norway

**Keywords:** parenting stress, pregnancy, attachment style, adverse childhood experiences, antenatal cumulative risk, infant temperament

## Abstract

Parenting stress can influence caregiving behavior negatively, which in turn may harm children’s development. Identifying precursors of parenting stress, preferably beginning during pregnancy and throughout the first year of life, is therefore important. The present study aims to provide novel knowledge on this issue through a detailed examination of the association between maternal attachment style and later parenting stress. Moreover, we examine the role of several additional risk factors, specificially the mothers’ own adverse childhood experiences (ACE), as well as infants’ temperamental characteristics. Data from a community based longitudinal study of 1,036 Norwegian mothers, collected during pregnancy and 12 months after childbirth, were used. Results showed that attachment style in pregnancy predicted parenting stress 1 year after birth. In addition, it was demonstrated that the mothers’ own ACEs predicted postnatal parenting stress, and that attachment style operated as a mediator of this association. A significant association between perceived infant temperament and parenting stress was also found. The study illustrates the importance of understanding the multifactorial antecedents of parenting stress. The results may inform early intervention efforts aimed at supporting mothers and their partners in the potentially difficult transition period around childbirth.

## Introduction

Parenting stress arises when the parent-child system is under strain. Such stress typically occurs when there is a mismatch between parents’ perception of available psychological and family resources and the demands of parenthood (Abidin, 1995; Deater-Deckard et al., 1998). According to Abidin (1995) parenting stressors are multidimensional, and can be organized in three major domains; parent characteristics, life stress and socio-demographic factors, and child characteristics. The present study aims to provide a better understanding of this conception through a detailed examination of the association between antenatal maternal attachment style and later parenting stress. In addition, we examine the association of several other potential risk factors for parenting stress, including mothers’ own adverse childhood experiences (ACE) and child temperament (Bates et al., 2012).

Previous research has suggested that parenting stress negatively influences parenting behavior, which in turn has been found to affect children’s development (Crnic et al., 2005). For example, it has been shown that maternal parenting stress shortly after birth is associated with an increased risk of perceiving the infant as difficult (Mäntymaa et al., 2009). Further, there is an association between parenting stress in the preschool years and child behavior problems in early and middle childhood (Neece et al., 2012). As a result, parenting stress has been considered a salient risk factor for aberrant child development (Abidin, 1992, 1995; Östberg and Hagekull, 2000; Mäntymaa et al., 2009). Even though many postnatal risk factors of parenting stress are well researched (e.g., Abidin, 1992, 1995; Östberg and Hagekull, 2000; Mäntymaa et al., 2009; Neece et al., 2012), antenatal risk factors have scarcely been investigated. Consequently, there is a need to identify precursors of parenting stress, preferably beginning during pregnancy and throughout the first year of life, in order to prevent possible harmful spirals of child development.

Becoming a mother often brings about personal adjustments and alterations in the individual’s self-identity and family relations, thereby leading to new attachment-related experiences (Stern, 1995; Earle, 2000; Ikeda et al., 2014). The quality of the mother’s representations of attachment relationships is an important factor to consider for maternal adaptation, because insecure maternal attachment has been found to influence parenting behavior negatively (Biringen et al., 2000; DeOliveira et al., 2005; Riva Crugnola et al., 2013), and to increase stress in the parenting role and in the parent–child relationship (Jones et al., 2015).

Bowlby (1988) suggested that attachment patterns reflect working models of the self and the other, representing both sides of the relationship. Such working models are formed already in the first years of life, based on actual interactions with the child’s primary caregivers. An infant who receives sensitive and responsive care from the caregiver is prone to form internal working models of the self as a person worthy of love and care, and of the other (the attachment figure) as someone who can be relied on when the attachment system is activated and emotional needs arise (cf. Bretherton and Munholland, 2008). Bowlby (1988) hypothesized that internal working models are the mechanism by which early attachment experiences influence functioning in close relationships later in life.

Attachment representations in adulthood have traditionally been measured with the Adult Attachment Interview (AAI), assessing individuals’ current “state of mind with respect to attachment” (Hesse, 2016). However, in the last decades self-report questionnaires, assessing adults’ underlying characteristic attachment style within a dimensional framework (e.g., Brennan et al., 1998), have been increasingly used when investigating the links between parental attachment representations and parenting stress (Jones et al., 2015). In contrast to the AAI, the most commonly used self-report measures of attachment styles, such as the Experiences in Close Relationships Scale (ECR, Brennan et al., 1998), do not focus on earlier childhood relationships with primary caregivers, but rather on more recent experiences in close relationships, such as partner relationships (Jones et al., 2015). In concordance with Bowlby (1982) notion of generalized internal working models, based on experiences in multiple relational contexts over time, the concept is thought to reflect global internal representations of the self and others in close relationships in general (Shaver and Mikulincer, 2004; Stern et al., 2018).

Adult attachment styles are often conceptualized as having two underlying dimensions; attachment-related avoidance and attachment-related anxiety (see Shaver and Mikulincer, 2002). An insecure avoidant attachment style is characterized by dismissing attachment-related issues, and an affect-regulating strategy involving deactivating the attachment system when in distress (i.e., keeping distance to attachment figures). In contrast, an anxious attachment style is characterized by a preoccupation with relationship-related worries, and an affect-regulating strategy of hyperactivating the attachment system when experiencing negative emotions (i.e., attempts to elicit emotional support through minimizing the distance to attachment figures; clinging and/or controlling responses) (Shaver and Mikulincer, 2002).

In their review of the literature on attachment style and parenting, Jones et al. (2015) identified 11 studies reporting significant associations between adult attachment style and parenting stress. Most of these studies concluded that both attachment-related avoidance and attachment-related anxiety are related to a higher degree of parenting stress. However, only a few studies have examined this issue longitudinally from pregnancy on (e.g., Rholes et al., 2006; Trillingsgaard et al., 2011; Mazzeschi et al., 2015). Rholes et al. (2006) assessed parents’ attachment style 6 weeks before birth and the experience of parenting stress when their infants were 6 months old. They found that more avoidant parents perceived parenting as more stressful than less avoidant parents, suggesting that this association might come about because avoidant individuals may have less of a desire to become parents, more depressive symptoms and less satisfying couple relationships, and that these factors mediate the outcome of increased parenting stress.

In another study (Mazzeschi et al., 2015) it was found that attachment-related anxiety was associated with parenting stress, when children were 3 months old, to a higher degree than attachment-related avoidance, whereas in a study by Trillingsgaard et al. (2011) both attachment-related avoidance and anxiety, assessed during pregnancy, were associated with parenting stress 1 year postpartum. It should be noted that two of these studies, (Rholes et al., 2006; Mazzeschi et al., 2015) had relatively small samples, and that they did not investigate important factors such as the mothers’ own adversities during childhood or the contribution of perceived infant behavioral characteristics on mothers’ experiences of parenting stress.

Adverse childhood experiences among parents have been shown to influence later parental emotional functioning and parenting stress (Steele et al., 2016). Such experiences are related to severe discord in the parents’ family of origin, and include for example, having had a parent with a mental disorder or using substances; having experienced physical, sexual or emotional abuse as a child; or having been exposed to severe abuse or neglect (Felitti and Anda, 2010). Studies have shown that ACEs are associated with later problems in social, emotional and cognitive domains, including compromised mental health, difficult romantic relationships, and insecure attachment styles, all of which may be related to aberrant parenting behavior and stress in the parenting role (Felitti et al., 1998; McCarthy and Taylor, 1999; Chapman et al., 2004; Felitti and Anda, 2010; McCarthy and Maughan, 2010; Murphy et al., 2014; Steele et al., 2016).

However, the mechanisms linking ACEs with parenting stress years later need to be elucidated. Possibly, experiencing severe adversity during childhood may contribute in shaping insecure attachment styles, through internal working models. Attachment styles have been shown to be more mallable in early childhood years, and become more stable as the child grows older (Fraley and Roisman, 2018), indicating the importance of early childhood experiences. In light of this, it is conceivable that attachment styles may operate as a mediator through which ACEs are associated with parenting stress.

When investigating how maternal attachment style may prospectively be related to parenting stress, it is also important to consider a multitude of other risk factors, such as maternal life stress, and demographic and perinatal risk factors (e.g., Matvienko-Sikar et al., 2017). It has been shown that maternal mental health factors, such as ante- and postpartum depression and anxiety, may prospectively predict parenting stress (Misri et al., 2010; Riva Crugnola et al., 2016; Huizink et al., 2017; Rollè et al., 2017). However, studies have shown that multiple risk exposure, in which the cumulative effect of risk factors are considered, exceeds the adverse developmental impacts of singular exposures (Sameroff et al., 1987; Trentacosta et al., 2008). Therefore, instead of assessing the importance of single risk factors, in the present study we chose the approach to consider how a series of events may have a cumulative effect.

Moreover, in line with a transactional model of child development (Sameroff, 2009), there is a need for more knowledge about how perceived infant characteristics may add to the effect of maternal attachment style on parenting stress (Mäntymaa et al., 2009). This perspective has been lacking in previous studies of the link between attachment style and parenting (Jones et al., 2015). Some infant characteristics can act as significant stressors affecting parenting capacity (Papoušek and von Hofacker, 1998). Such characteristics often include infant fussiness and negative emotionality, which can be expressions of temperamental dispositions (Bates et al., 1979; Chess and Thomas, 1999).

Much research has focused on the role of the child’s negative emotionality for parenting stress (e.g., Mäntymaa et al., 2009; Siqveland et al., 2013; Prino et al., 2016). One should also consider other aspects of temperament, such as how the infant adapts to changing situations, the degree of persistence in continuing activities, and the predictability of natural functions, including sleep and mealtime regularities. Infants with low adaptability, persistence, or regularity might be perceived as difficult and be a source of parenting stress, whereas highly adaptable, persistent, and regular infants could be perceived as favorable by their parents, and thus serve as a buffer against parenting stress.

To sum up, even though research has shown that attachment style is related to parenting stress, there are few longitudinal studies with large sample sizes that examine how attachment style among pregnant women predicts subsequent parenting stress, and whether maternal attachment style may be conceived as a mediator in the relation between ACEs and parenting stress. Further, there is a need for studies examining how infant characteristics, such as perceived temperament, are associated with parenting stress, and how aspects of infant temperament may moderate the link between maternal attachment style and parenting stress.

### Aims and Hypotheses

This study has four aims:

First, to investigate the prospective association between maternal attachment styles during pregnancy and parenting stress when infants are 12 months old, while taking into account the contribution of other factors, including a variety of maternal risk factors. Second, to investigate whether mothers’ own recollected ACEs are related to parenting stress and, if so, whether this association is mediated by attachment style. Third, to investigate the association between perceived infant temperament and maternal parenting stress at 12 months, while accounting for the influence of several maternal and infant risk factors. And, finally, to investigate whether the association between attachment style and parenting stress is moderated by perceived child temperament.

Based on previous research, we hypothesized that attachment style during pregnancy would bear a relation to parenting stress 12 months after birth. We also hypothesized that maternal attachment style would mediate the pathway between maternal ACEs and later parenting stress. We further hypothesized that the association between attachment style and parenting stress is moderated by how mothers rate infant temperament. In particulary, we expected that this association might only be evident at lower levels of infant adaptability, persistence, and regularity.

## Materials and Methods

This paper reports data from 1,036 families participating in the multisite. prospective, longitudinal *Little in Norway* study (Skjothaug et al., 2015; Fredriksen et al., 2017; Olafsen et al., 2018). Data used in the present report were collected from mothers during pregnancy (T1) and again at 12 months after childbirth (T2).

### Procedure

The enrollment took place from September 2011 to October 2012. All pregnant women receiving routine prenatal care at well-baby clinics at nine geographically diverse sites across Norway were invited by midwives to participate. The clinics were chosen after considering demographic characteristics and size of the population to include participants from both cities and rural districts with a wide distribution of socioeconomic conditions.

Each participant was informed about the purpose of the study. Confidentiality was assured, and it was emphasized that participation was voluntary and they could withdraw at any time. At each site, one public health care nurse was trained as a research assistant to administer the questionnaire forms.

During pregnancy (T1) we assessed maternal attachment style, as well as the occurrence of adverse experiences in the mothers’ own childhood. A maternal cumulative risk index was also computed, based on eight antenatal maternal risk indicators. When the child was 12 months old (T2), maternal perception of child temperament, as well as experience of stress in the parenting role and in the parent–child relationship, were assessed.

The study protocol and the assessment procedures were reviewed and approved by the Norwegian Regional Committees for Medical and Health Research Ethics, reference number 2011/560.

### Participants

Initially, 1,041 pregnant women consented to participate in the study; five women later withdrew their consent, leaving 1,036 (99.5%) participating women. No exclusion criteria were applied. The response rate was 50.7% (Fredriksen et al., 2017).

We used *N* = 1,036 persons in our analyses before childbirth (at T1). The sample consisted of 10 twin pairs, and the second twin of each pair was excluded. Of this sample, most women (*n* = 973, 93.9%) were of Norwegian origin. At enrollment mean maternal age was 30 years (range: 17–43, *SD* = 4.8). Most participants were either married or cohabiting (*n* = 994, 95.9%), with only a small fraction being single (*n* = 26, 2.5%) or divorced/separated (*n* = 2, 0.2%), or having missing information on marital status (*n* = 14, 1.4%). Most of the participants (*n* = 799, 77.1%) had a college or university degree. The highest completed education of the remaining participants was high school (*n* = 205, 19.8%) or lower (*n* = 32, 3.1%).

Pearson chi-square tests were performed for each of the study’s nine sites, comparing the educational level of participating mothers with that of women in the same age range and at the same geographical sites (figures obtained from Statistics Norway). Generally, there were significant differences (*p* < 0.001), with study participants having a higher educational level. Of the 1,036 women participating at T1, 744 mothers participated at 12 months after childbirth, T2 (71.8% response rate). Attrition analyses were conducted by means of independent samples *t*-tests. The analysis showed that the mothers who did not participate at T2 were included several days later in pregnancy (*M* = 169.86, *SD* = 35.08) than the women who did participate at T2 [*M* = 162.14, *SD* = 34.10; *t*(1034) = 3.21, *p* = 0.001], and scored somewhat higher on the cumulative risk index in pregnancy (*M* = 0.99, *SD* = 1.00) than the mothers who did participate at T2 [*M* = 0.85, *SD* = 0.99; *t*(1034) = 2.05, *p* = 0.041]. They also reported more frequent experiences of ACEs (*M* = 0.93, *SD* = 1.64) than participating mothers [*M* = 0.68, *SD* = 1.25; *t*(1033) = 2.73, *p* = 0.006]. A Chi-square test indicated a significant association between attrition and level of education, (χ^2^ = 23.79, *p* < 0.05). Attrition was not related to maternal age, parity, if women ever had a psychiatric consultation, or attachment styles (*p* > 0.05).

### Measures

#### Antenatal Measures (T1, Pregnancy)

##### Maternal attachment style

Attachment style was measured at T1 by the Experiences in Close Relationships (ECR) Scale (Brennan et al., 1998). The ECR Scale (Brennan et al., 1998) is among the most commonly used self-report measures designed to assess the dimensions of adult attachment styles in relationships and the scale has been used in numerous studies focusing on the links between parents’ self-reported attachment styles and parenting behaviors, emotions and cognitions (Jones et al., 2015; Stern et al., 2018). The ECR Scale was designed to assess the pattern of adult attachment styles in relationships generally, rather than focusing on romantic partner relationships specifically. The general ECR version consists of two dimensions, avoidance and anxiety, measured by 18 items each. The two scales have been found to be reliable both concerning internal-consistency and test–retest, and to have high construct, predictive, and discriminant validity (Crowell et al., 1999). A previous Norwegian study of a population based sample found the psychometric properties of the ECR to be satisfying when used in a Norwegian context (Olssøn et al., 2010). Response options range from 1 (*strongly disagree*) to 7 (*strongly agree*). Sum scores were computed, with higher scores reflecting greater levels of insecure attachment within each relationship’s domain (range 18–126 on each subscale). Mikulincer and Shaver (2004) reported Cronbach alpha coefficients near or above 0.90 and test–retest coefficients between 0.50 and 0.75. In the present study, Cronbach’s alphas were 0.88 and 0.89 for the anxiety and avoidance subscales, respectively.

##### Adverse childhood experiences

Adverse childhood experiences were assessed retrospectively in pregnancy using the Adverse Childhood Experiences Scale (ACE Scale; Felitti and Anda, 2010). This scale consists of ten questions about possible adverse experiences prior to age 18, such as physical or sexual assault, major separation or loss, and parental mental illness or drug abuse. In the scoring system, the presence of each type of the ten possible experiences counts as one point. Dube et al. (2004) assessed the test-retest reliability of the ACE scale and found good to moderate reliability of the subscales. Since the ACE scale addresses diverse adverse childhood events, it is not likely that there is a unified underlying conceptual dimension for this measure; thus Cronbach’s alpha is not reported.

##### Maternal cumulative risk during pregnancy

As alluded to in the introduction, studies have shown that multiple risk exposure exceeds the adverse developmental impacts of singular exposures (Sameroff et al., 1987; Trentacosta et al., 2008). Therefore, instead of assessing the importance of single risk factors, the approach to consider how a series of events may have a cumulative effect was chosen. A cumulative risk index comprising eight antenatal risk indicators was constructed. Each item was scored optimal (0) or non-optimal (1) indicating risk, yielding a cumulative risk score. The eight items were as follows: (1) Intention to cohabit with the child’s father. No indicates risk. (2) Was the pregnancy desired? No indicates risk (3) Pregnancy-related anxiety assessed by the Pregnancy Related Anxiety Questionnaire-Revised (PRAQ-R; Huizink et al., 2004). PRAQ > 30 indicates risk. (4) Antenatal depression assessed by the Edinburgh Postnatal Depression Scale (EPDS; Cox et al., 1987). EPDS ≥ 10 indicates risk. (5) Daily smoking in pregnancy. Yes indicates risk. (6) Life stress assessed by the Parenting Stress Index, Life Stress subscale (PSI LS; Abidin, 1995). PSI LS ≥ 17 indicates risk. (7) Pregnancy risk drinking assessed by the Tolerance, Worried, Eye-opener, Amnesia, Cut-down (TWEAK) instrument (Russell, 1994). TWEAK ≥ 2 indicates risk. (8) Use of prescribed medication in pregnancy. Yes indicates risk.

In addition to the risk index, the following covariates were included: previous mental health consultations, maternal education, number of previous children, maternal age, days of gestation at inclusion at T1, infant gender, infant birthweight, and gestational age.

#### Postnatal Measures (T2, 12 Months After Birth)

##### Parenting stress

Stress in the parenting role and in the parent–child relationship was assessed at T2 by the Parenting Stress Index (PSI; Abidin, 1995). The PSI is a self-report questionnaire that provides an estimate of stress experienced by parents. The instrument contains a Child Domain and a Parent Domain. It was standardized for use with parents of children ranging from 1 month to 12 years of age, and has acceptable reliability and validity (Abidin, 1995). The instrument comprises 101 items, each rated on a 5-point scale (from *strongly agree* to *strongly disagree*). The Child Domain reflects how parents perceive their child and contains six subscales: Distractibility/Hyperactivity (9 items), Adaptability (11 items), Parent Reinforcement (6 items), Demandingness (9 items), Mood (5 items), and Acceptability (7 items). The Parent Domain consists of items related to parental coping and the parenting role; it comprises seven subscales: Competence (13 items), Isolation (6 items), Attachment (7 items), Health (5 items), Role Restriction (7 items), Depression (9 items), and Spouse (7 items). Higher scores indicate more stress. Cronbach’s alphas were 0.81 for the Child Domain and 0.84 for the Parent Domain.

##### Child temperament

The Cameron-Rice Infant Temperament Questionnaire (CRITQ; Cameron et al., 2013) assesses infants’ temperament characteristics during the first year of life, based on the framework provided by the New York Longitudinal Study (NYLS; Chess and Thomas, 1984). The original CRITQ comprises 46 items and is a shortened and adapted version of the Carey Temperament Scales (CTS; Carey and McDevitt, 1978). The CRITQ was developed for individualized parent guidance within US Health Maintenance Organizations, and the scale has recently been examined for psychometric properties in a Norwegian context (Olafsen et al., 2018). The three dimensions Adaptability, Persistence, and Regularity had unidimensional factor structures in confirmatory factor analyses (CFA) and were focused in the present study. *Adaptability* has 3 subscales (*adaptability to restrictions and intrusions, adaptability to transitions, adaptability to changes*) with a total of 10 items; *persistence* has 5 items and no subscales; *regularity* has 3 subscales (*regularity sleep, regularity feeding, regularity activity*) with a total of 8 items. The questionnaire uses a 6-point Likert scale (1 = *almost never* to 6 = *almost always*). Cronbach’s alphas for the three scales ranged from 0.56 for *adaptability*, to 0.66 for *persistence* and 0.67 for *regularity* (cf. Olafsen et al., 2018). To reduce response ambiguity, the questionnaire asks about specific behaviors. The scale was translated into Norwegian and back-translated into English.

### Statistical Analyses

First, descriptive statistics and inter-correlation analyses were conducted. Inter-correlations were computed using Pearson product-moment correlation coefficients for continuous variables and Spearman rank order correlation coefficients for categorical variables. Then, latent factors of the outcome variables were modeled by CFA in a structural equation (SEM) framework. The latent factors of the PSI Child and Parent Domains, respectively, were then included as the outcome variables in SEM models with attachment anxiety and avoidance as predictors, adjusting for an extensive number of covariates. Next, using SEM, we conducted multiple parallel mediation analyses to test the simultaneous effects of anxious and avoidant attachment as potential mediators on the relation between ACE and parenting stress.

Moreover, interaction analyses were conducted by including attachment, child temperament, and the product term of both variables as predictors in SEM models with the latent factors of the PSI Child and Parent Domains as outcomes. Robust maximum likelihood estimation procedures were employed to account for potential non-normality. Missing data were handled by the full information maximum likelihood procedure, thereby providing missing data routines that are considered to be adequate (Schafer and Graham, 2002). As recommended (Hayes, 2013), bias-corrected bootstrap confidence intervals were estimated in all mediation analyses using 5,000 bootstrap samples. Significant results were determined by *p <* 0.05. Descriptive statistics and correlations were estimated using SPSS Version 22. The statistical program Mplus 7.4 was used for all other analyses.

## Results

### Descriptive Statistics

Table 1 lists the descriptive statistics and correlations for all variables used in the study.

**Table 1:** Means, standard deviations, and correlation matrix of all measures.

Measure (range)	Mean	*SD*	1	2	3	4	5	6	7	8	9	10	11	12	13	14	15	16
(1) PSI Child Domain (52–141)	85.87	14.34					.											
(2) PSI Parent Domain (61–199)	108.02	20.66	0.60^∗∗^															
(3) ECR Avoidance (18–90)	30.05	12.68	0.21^∗∗^	0.35^∗∗^														
(4) ECR Anxiety (18–114)	44.23	17.18	0.30^∗∗^	0.43^∗∗^	0.33^∗∗^													
(5) ACE (0–9)	0.75	1.37	0.12^∗∗^	0.16^∗∗^	0.25^∗∗^	0.24^∗∗^												
(6) Risk Index (0–6)	0.89	0.99	0.16^∗∗^	0.21^∗∗^	0.27^∗∗^	0.38^∗∗^	0.21^∗∗^											
(7) Maternal age (17–43)	30.11	4.79	−0.04	0.02	0.06^∗^	−0.12^∗∗^	−0.05	−0.26^∗∗^										
(8) Previous children (0–5)	0.70	0.78	−0.11^∗∗^	0.00	0.10^∗∗^	−0.09^∗∗^	0.05	−0.15^∗∗^	0.35^∗∗^									
(9) Maternal education (1–4)	3.15	0.84	0.05	−0.03	−0.12^∗∗^	−0.08^∗^	−0.10^∗∗^	−0.27^∗∗^	0.36^∗∗^	−0.03								
(10) Mental health consult. (0–1)	0.17	0.37	0.10^∗∗^	0.13^∗∗^	0.10^∗∗^	0.20^∗∗^	0.22^∗∗^	0.11^∗∗^	−0.03	−0.03	−0.05							
(11) Days of gestation (55–238)	164.32	34.54	0.03	−0.01	−0.03	0.04	−0.01	−0.06	0.14^∗∗^	0.00	0.11^∗∗^	0.01						
(12) Infant birth weight (kg) (0.64–4.94)	3.53	0.54	−0.10^∗∗^	−0.03	−0.02	−0.04	0.02	−0.03	0.02	0.11^∗∗^	−0.03	−0.02	0.04					
(13) Infant GA (weeks) (24–43)	39.48	1.90	−0.11^∗∗^	−0.02	−0.03	−0.02	0.02	−0.03	0.00	−0.07^∗^	−0.01	0.01	0.09^∗∗^	0.59^∗∗^				
(14) Infant gender (1–2)	1.48	0.50	0.01	0.03	−0.03	0.01	−0.01	−0.03	−0.00	0.01	0.00	−0.04	−0.03	−0.07^∗^	0.04			
(15) Infant persistence (12–100)	62.42	16.56	−0.30^∗∗^	−0.16^∗∗^	0.00	−0.03	0.04	0.05	−0.02	−0.02	−0.06	−0.04	−0.05	0.06	0.10^∗∗^	−0.04		
(16) Infant regularity (0–68.57)	24.30	12.56	−0.29^∗∗^	−0.28^∗∗^	−0.13^∗∗^	−0.15^∗∗^	0.02	−0.08^∗^	0.04	0.05	0.07	0.01	−0.03	0.05	0.06	0.03	0.14^∗∗^	
(17) Infant adaptability (22–100)	68.80	11.79	−0.47^∗∗^	−0.33^∗∗^	−0.08^∗^	−0.18^∗∗^	−0.08^∗^	−0.06	−0.02	−0.08^∗^	−0.01	−0.08^∗^	−0.05	0.07	0.04	−0.02	0.24^∗∗^	0.32^∗∗^

*PSI Child Domain, Parenting Stress Index Child Domain; PSI Parent Domain, Parenting Stress Index Parent Domain; ECR Avoidance, Experiences in Close Relationships Avoidance domain; ECR Anxiety, Experiences in Close Relationships Anxiety domain. Level of maternal education: 1 = 9–10 years or less, 2 = High school, 3 = 1–3 years of college/university education 4 = 4 years or more of university education. Mental health consult., Whether one ever had a mental health consultation. Days of gestation, Days of gestation at inclusion at T1; GA, Infant gestational age. Infant gender: 1 = boys (52%), 2 = girls (48%). Infant persistence, infant regularity and infant adaptability = 3 dimensions from The Cameron-Rice Infant Temperament Questionnaire (CRITQ). Pearson product-moment correlation coefficients were used for continuous variables, and Spearman rank order correlation for categorical variables. ^∗^p < 0.05. ^∗∗^p < 0.01.*

The table shows that the mean value of the cumulative risk index (0.89) and the mean values of the ECR avoidance and anxiety (30.05 and 44.23, respectively) were all at the lower end of the scales, as expected in a community-based sample such as the present one. More than half of the women (*n* = 569, 54.9%) had no previous child, whereas 345 women (33.3%) had one previous child, 103 women (9.9%) had two previous children, and 19 women (1.9%) had three or more previous children. At T2, PSI Child Domain scores ranged from 52 to 141 (mean 85.87), and PSI Parent Domain scores from 61 to 199 (mean 108.02).

Further, the table shows that ECR avoidance and ECR anxiety were both significantly and positively related to the outcome variables (PSI Child and Parent Domain), and significantly and negatively related to infant adaptability and infant regularity. Moreover, ACE was significantly and positively related to ECR avoidance and anxiety, and to the PSI Child and Parent Domain.

The cumulative pregnancy risk index bore significant and positive associations with the PSI Child and Parent Domains, ECR avoidance, ECR anxiety, ACE, and infant regularity at 12 months. The risk index had significant and negative associations with maternal age, number of previous children, level of education and having previous mental health consultations. Previous mental health consultations were further significantly and positively related to ECR avoidance and ECR anxiety, PSI Child and Parent Domains and ACE, while infant gestational age and birth weight bore negative and significant associations with the PSI Child Domain. It should be noted that the correlations between the independent variables were moderate to low; thus, there should be no concern regarding their use as separate independent variables in the regression analyses.

### Structural Equation Models of Antenatal Maternal Measures, Postnatal Measures, and Maternal Parenting Stress

Initially, a CFA was conducted with PSI subscales as indicators to model PSI Child and Parent Domains as latent factors. More specifically, the subscales distractibility/hyperactivity, adaptability, reinforces parent, demandingness, mood, and acceptability were modeled to load on the Child Domain, whereas competence, isolation, attachment, health, role restriction, depression and spouse were modeled to load on the Parent Domain. Both factors were included in one structural equation model and the two latent factors were allowed to correlate. The fit indices indicated a somewhat low fit of data to the model; CFI = 0.91, TLI = 0.89, RMSEA = 0.078 [90% CI: 0.070,0.086]. The factor loadings were all substantial (standardized factors loadings > 0.50), with the exception of the parent reinforcement subscale that fell below 0.50. The model was thus modified by removing this subscale as an indicator of the Child Domain. The fit of the modified model was substantially improved by removing the subscale; CFI = 0.94, TLI = 0.93, RMSEA = 0.068 [90% CI: 0.059,0.077], and these latent factors were used in subsequent analyses.

SEM analyses were conducted with the latent factors of PSI Child and Parent Domains as outcome variables. In a first step, only ECR avoidance and ECR anxiety were included to predict the latent parenting stress factors. As shown in Table 2, Model 1, both partner attachment variables significantly predicted parenting stress related to the Child Domain as well as the Parent Domain.

**Table 2:** Structural equation models with PSI Parent and Child Domains as outcomes.

	PSI Parent Domain			PSI Child Domain		
	B	*t*	*P*	B	*t*	*P*
**Model 1**						
ECR Avoidance	0.25	5.63	<0.001	0.13	2.77	0.006
ECR Anxiety	0.40	10.55	<0.001	0.27	6.50	<0.001
**Model 2**						
ECR Avoidance	0.23	5.20	<0.001	0.15	3.24	0.001
ECR Anxiety	0.40	10.22	<0.001	0.24	5.37	<0.001
ACE	0.01	0.26	0.795	0.03	0.65	0.514
Cumulative risk	0.02	0.39	0.696	0.04	0.69	0.489
Maternal age	0.06	1.44	0.150	0.00	0.00	0.999
Previous children	−0.01	0.23	0.816	−0.10	2.36	0.018
Maternal education	−0.02	0.40	0.688	0.09	2.01	0.045
Mental health consultation	0.04	0.85	0.395	0.01	0.32	0.750
Days of gestation at T1	0.00	0.11	0.914	0.02	0.38	0.701
**Model 3**						
ECR Avoidance	0.21	4.48	<0.001	0.11	2.80	0.005
ECR Anxiety	0.35	8.27	<0.001	0.15	3.44	0.001
ACE	0.02	0.44	0.661	0.04	1.04	0.298
Cumulative risk	0.03	0.66	0.511	0.04	0.95	0.341
Maternal age	0.07	1.69	0.091	0.01	0.22	0.822
Previous children	−0.02	0.44	0.661	−0.13	3.24	0.001
Maternal education	−0.03	0.63	0.528	0.07	1.57	0.116
Mental health consultation	0.03	0.83	0.406	0.00	0.00	0.998
Days of Gestation at T1	−0.02	0.63	0.526	0.00	0.23	0.819
Infant birth weight	0.01	0.18	0.857	0.00	0.08	0.701
Infant gestational age	0.00	0.06	0.952	−0.08	1.99	0.047
Infant gender	0.02	0.66	0.507	0.01	0.38	0.701
Infant persistence	−0.09	2.36	0.018	−0.20	5.22	<0.001
Infant regularity	−0.14	3.49	<0.001	−0.10	2.50	0.013
Infant adaptability	−0.19	4.46	<0.001	−0.43	10.60	<0.001

*PSI Child Domain, latent factor of Parenting Stress Index Child Domain; PSI Parent Domain, latent factor of Parenting Stress Index Parent Domain; ECR Avoidance, Experiences in Close Relationships Avoidance subscale; ECR Anxiety, Experiences in Close Relationships Anxiety subscale. β, standardized regression coefficient.*

In the next step, ECR avoidance and ECR anxiety were entered together with ACE and the cumulative pregnancy risk, maternal age, number of previous children, maternal education, previous mental health consultations, and gestation week at inclusion at T1 (see Model 2, Table 2). The results show that, when adjusting for all covariates, both attachment variables still predicted parenting stress related to the Child Domain as well as the Parent Domain.

In a third step, child temperament (i.e., infant persistence, regularity and adaptability), infant birth weight, gestational age, and infant gender were additionally entered as predictors. As shown in Table 2, Model 3, the association between partner attachment (ECR avoidance and anxiety) and parenting stress in both the child and the parental domains remained significant in the last model. That is, high scores on ECR anxiety predicted uniquely and significantly elevated levels of parenting stress in both the Child and Parent Domains, even when controlling for a comprehensive number of covariates. Similar results were found for the ECR avoidance scale, with significant relations to parenting stress in both the Child and Parent Domains. Results further showed that all three infant temperament dimensions were significantly associated with both the PSI Child and Parent Domains (*p* < 0.05). Overall, most other predictor variables had small or no significant association with the two outcome variables in all SEM analyses (see Table 2).

### Mediation Analyses With Attachment Styles as Mediators

We also examined the associations between ACEs and the PSI Child and Parent Domains in more detail. First, structural equations were modeled to examine the associations between ACE and the latent PSI factors while accounting for covariates. Initially, ECR avoidance and ECR anxiety were not included in the analyses. Results showed that ACE significantly predicted both the Parent Domain (β = 0.10, *p* = 0.032) and the Child Domain (β = 0.08, *p* = 0.040). Second, mediation analyses were conducted to test the effects of anxious and avoidant attachment (ECR avoidance and ECR anxiety) simultaneously as potential mediators between ACE and the PSI Child and Parent Domains, respectively.

**FIGURE 1:**
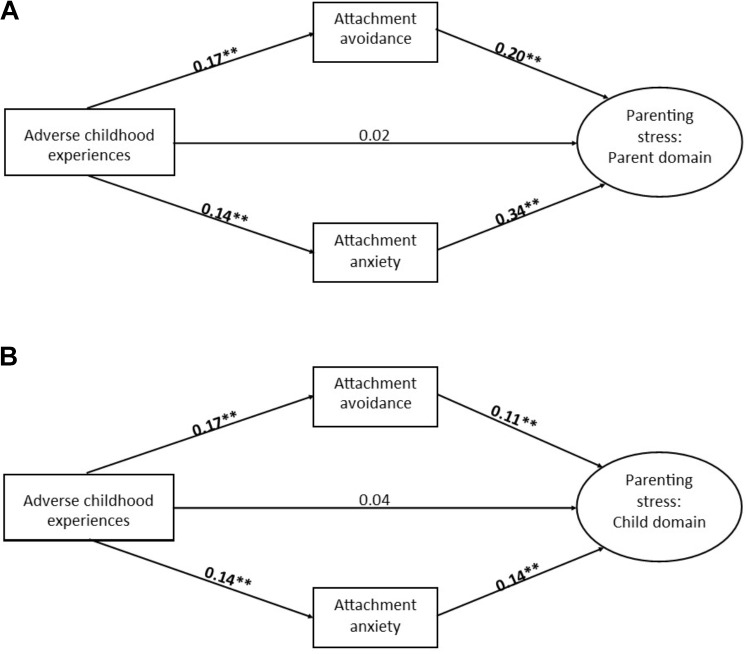
Graphical display of multiple mediation in a structural equation modeling framework. The dependent variable and mediators are controlled for all covariates. Squares indicate manifest variables and circles indicate latent factors. Standard errors were estimated by means of bootstrapping with 5,000 bootstrapping samples. Standardized coefficients are presented. Panel **(A)** shows results of mediation analyses with parenting stress – Parent Domain as outcome. The total effect from adverse childhood experiences on the Parent Domain was β = 0.10, *p* = 0.032. The indirect effect via attachment avoidance was β = 0.04, *p* < 0.001. The indirect effect via attachment anxiety was β = 0.05, *p <* 0.001. Panel **(B)** shows mediation analyses with parenting stress – Child Domain as outcome. The total effect of adverse childhood experiences on the Child Domain was β = 0.08, *p* = 0.040. The indirect effect via attachment avoidance was β = 0.02, *p* = 0.015. The indirect effect via attachment anxiety was β = 0.02, *p* = 0.007. ^∗∗^*p* < 0.01.

As shown in Figure 1A, ACE was found to have a significant indirect effect on the PSI Parent Domain both through ECR avoidance and ECR anxiety. Further, as shown in Figure 1B, ACE was also found to have a significant indirect effect on the PSI Child Domain both through ECR avoidance and through ECR anxiety.

### Interaction Analyses With Child Temperament as Moderator

Finally, interaction terms were included in a series of SEM models to test possible moderating effects of child temperament characteristics on the association between partner attachment styles and the latent factors of parenting stress. Specifically, interaction terms were formed by the product of attachment styles and temperament characteristics, with the latent factors of parenting stress as outcomes. Results showed that none of the interaction terms in the 12 interaction analyses that were conducted (2 latent parenting stress domains × 2 attachment styles × 3 temperamental characteristics) were significant (*p* > 0.05).

As a test of robustness of our findings, all analyses were re-run with the parent reinforcement subscale included as an indicator of the latent factor of PSI child domain, in keeping with the recommendations in the PSI manual (Abidin, 1995). Moreover, we also re-ran all analyses with the PSI Child and Parent Domains as manifest variables. There were no substantial change in results with either of these alternative approaches.

## Discussion

The first aim of this study was to determine the predictive relation of maternal attachment style assessed during pregnancy to later parenting stress, while the second was to investigate the possible mediating role of pregnant women’s attachment style in the maternal ACEs – parenting stress pathway. After we accounted for the influence of several maternal and infant risk factors, results showed that both attachment-related avoidance and attachment-related anxiety were significantly related to parenting stress at 12 months. Moreover, attachment style accounted for much of the association between maternal ACEs and later parenting stress, suggesting a mediation effect. The third aim of the study was to examine the concurrent association between perceived infant temperament and maternal parenting stress at 12 months. Results showed that all of the three temperament dimensions had significant associations with both the Child and the Parent Domains of the parenting stress scale. The final aim was to investigate whether the association between attachment style and parenting stress was moderated by child temperament; however, no significant moderator effects were detected.

### Antenatal Attachment Style and Later Parenting Stress

Our first hypothesis, namely that antenatal attachment style would bear a relation to parenting stress 12 months after birth of the child, was supported. Further, the results lend support to previous research by Rholes et al. (2006) and Mazzeschi et al. (2015), who found that secure mothers are more able to cope with pregnancy and the transition to parenthood than are insecure mothers. However, these studies did not follow their participants longer than 3 and 6 months after birth, respectively. As the study by Trillingsgaard et al. (2011), the present work extends the predictor–outcome interval considerably, showing that attachment style, assessed before birth, may still have an effect on parenting stress at 12 months after birth, at a vulnerable time when the mother–infant attachment pattern is being consolidated.

In the present study both attachment-related avoidance and attachment-related anxiety were associated with stress in the Parent as well as in the Child Domain. This is in accordance with results of Trillingsgaard et al. (2011) research. However, while Rholes and Simpson (2004) showed that the dimension of attachment avoidance was a strong predictor of later parenting stress, Mazzeschi et al. (2015) demonstrated that although both attachment dimensions were related to parenting stress, attachment-related anxiety was the most salient predictor. It should be noted that Rholes et al. (2006) argue that there is no evidence to suggest that the two dimensions have differential associations with the experience of parenting stress. This is in line with the review by Jones et al. (2015) where the authors suggest that avoidant and anxious attachment styles are about equally related to parenting stress, even though parents who are more anxious or more avoidant may differ in the strategies they use to handle the stress they are experiencing.

### Maternal Attachment Style as a Mediator of the Association Between Adverse Childhood Experiences and Parenting Stress

Our second hypothesis, that maternal attachment styles would serve a mediating role in the ACEs–later parenting stress pathway was also supported. Initial analysis showed that maternal ACEs were significantly associated with parenting stress, in both the Child and Parent Domains. This finding is in line with results from a study by Steele et al. (2016), although their findings were based on a much smaller, high-risk sample. When formal mediation analyses were applied in the present study, the association between ACEs and both the Parent Domain and the Child Domain of parenting stress were mediated by maternal attachment style. In a study by Murphy et al. (2014) an association between ACEs and later interview-based adult attachment classification (assessed by AAI), was reported. They found that as the number of ACEs increased, so did the probability of insecure or unresolved adult attachment classifications.

What does it mean psychologically to include the maternal attachment style as a mediator in the model? As Bowlby (1988) emphasized, attachment is hypothesized to be at least partly based on early relationship experiences, but it is also partly influenced by concurrent experiences. Thus, ACEs may lead to insecure internal working models of relationships to significant others, which render a mother more susceptible to developing a more avoidant or a more anxious attachment style (Mikulincer and Shaver, 2004). Insecure attachment styles may in turn have a negative impact on parenting behavior and parenting stress, as they may result in a non-optimal relationship with the father of the child, which again may prevent optimal interaction between parents when bringing up their child. Moreover, mothers’ insecure internal working models may also impede the developing of an optimal mother–child relationship, and such mechanisms may in turn increase parenting stress (Jones et al., 2015).

It should be noted that a negative relation between ACEs and later coping has also been documented among men. For example, Opacka-Juffry and Mohiyeddini (2012) found that ACEs are negatively related to oxytocin system functioning in adult men, suggesting increased susceptibility to stress. Moreover, it has been shown that ACEs among fathers bear a relation with depressive and anxious feelings during pregnancy (Skjothaug et al., 2015), as well as with parenting stress at 6 months (Skjothaug et al., 2018). Future studies should investigate whether fathers’ attachment style may be a mediating mechanism in the pathway between ACEs and later paternal stress, as was shown among mothers in the present study.

### Infant Temperament and Parenting Stress

Our third hypothesis, namely that parenting stress is influenced by concurrent infant behavioral characteristics as perceived by the mother, was also supported. This result is in accordance with a transactional model of child development (Sameroff, 2009), underscoring that child characteristics, and how they are perceived and interpreted by the parent, play an important role in how child and caregiving context mutually shape each other over time. While several previous studies have focused on temperamental characteristics such as negative emotionality and parenting stress (e.g., Mäntymaa et al., 2009; Siqveland et al., 2013; Prino et al., 2016), scant attention has been directed at dimensions such as adaptability, persistence and regularity, all important aspects of infant behavior and development (Olafsen et al., 2018).

The results of the present study show that each of these dimensions had a significant and negative association with the Child and Parent Domains of parenting stress. Infants with low adaptability, persistence, or regularity may need extra support in the form of routines, rituals, and other adjustments, which can fuel mothers’ fears about the infant’s behavioral functioning (Papoušek et al., 2008). Parents may need to invest more resources and might be more limited in their daily life due to less sleep and more worry about their children’s development. Thus, having an infant who is perceived as temperamentally difficult might be related to parenting stress, both in connection with the child’s behavioral characteristics and the parents’ adaptation to and coping with the parental role.

Although parents’ perceptions of their infants’ temperament are based on daily experiences, there is also an envelope of influences on how parents rate their infant’s characteristics emanating from outside the actual interactions (Bates and Bayles, 1984; Zeanah and Anders, 1987; Mebert, 1991). Several studies have shown that parental factors such as SES, anxiety, depression, and attachment style are related to perceived child temperament (Pesonen et al., 2004; Seifer et al., 2004). For example, Pesonen et al. (2004) reported that parental insecure attachment style was associated with concurrent perceptions of 6-month-old infant’s temperament as more fearful, more easily distressed and negatively reactive, illustrating that preexisting parental representations can feed perceptions of the infant, and thus lay the foundation for suboptimal interaction patterns and parenting stress.

### Infant Temperament as Moderator of the Association Between Attachment Style and Parenting Stress

Our final hypothesis that the association between maternal attachment styles and parenting stress would only be evident at lower levels of infant adaptability, persistence, and regularity was not supported, as no moderation effect of temperament was demonstrated. This suggests that maternal attachment style influences parenting stress, no matter how temperamentally difficult or easy the child may be.

It is possible that the timing of temperamental assessment in this study has played a role. For example, individual differences in level of irritability and frustration tolerance (Rothbart, 1986), and possibly also adaptability and regularity, are more pronounced at 5–6 months than at 1 year of age. Since temperament may change early in life (Rothbart and Bates, 2006), it may be that infant characteristics such as adaptability, persistence, and regularity could have played a moderating function if they had been assessed in the middle of the first year (cf. Bridgett et al., 2009).

### Strengths and Limitations

This study was based on longitudinal data from a large-scale community-based sample. Moreover, well-validated measures were used to assess both attachment style and parenting stress. In addition, we included several maternal pregnancy-related risk factors, negative events in the mothers’ own childhood, as well as infant perinatal factors, such as gestational age and birthweight, and concurrent infant temperament, and investigated their contribution to parenting stress at 12 months.

The study also has several limitations. The women who took part tended to have a somewhat higher educational level than what was common in the general population at the different sites. Even though the educational level of the participating mothers was controlled for, the impact of low education might have been different had it been more in line with that of the general population. Moreover, the mothers who did not participate at 12 months had a somewhat higher cumulative risk score in pregnancy than those who did participate at that point. However, appropriate statistical methods (i.e., full information maximum likelihood estimations) were used to minimize the effect of selective attrition.

The study is further limited by using mothers as single informants, as the statistical associations might be inflated by shared methods variance. Further, cognitive biases and inflexible response-tendencies may be introduced, influenced by defensiveness, self-protection, oversensitivity, or obliviousness, which might bring inaccuracy to the rating process (Reznick and Schwartz, 2001). This limitation needs to be taken into consideration when interpreting the results, although we emphasize that the main aim of the study was to focus specificially on mothers’ own experiences before and after childbirth.

Another related limitation is that the present study did not include fathers. Future studies should include fathers as well as mothers in order to study how the association between attachment style, mental health and parenting stress may differ between parents, and how one partner’s attachment style may moderate changes in the other partner’s attachment style across the transition to parenthood (Simpson et al., 2003; Simpson and Rholes, 2018).

Also, the measures were based on self-reports, rather than psychiatric evaluation, diagnoses, or attachment interviews. This issue applies particularly to our use of a self-report measure of attachment style. However, studies have shown that the ECR attachment scale has good reliability and validity (cf. Brennan et al., 1998; Fraley et al., 2000). Several studies have shown that self-report measures of adult attachment style relate to ways in which a person discusses close relationships, to observations of marital communication, to patterns of self-disclosure, and to seeking and providing social support under stressful conditions (cf. Bartholomew and Shaver, 1998). Further, it has been reported that the anxiety scale is associated with measures of anxiety and preoccupation with attachment issues, jealousy, and fear of rejection, and that the avoidance scale correlates highly with several other measures of avoidance and discomfort with closeness (Brennan et al., 1998).

The retrospective nature of the ACEs scale also represents a limitation since the validity of the scale might be questioned. However, longitudinal follow-up studies of adults whose childhood abuse was documented through records and interviews have consistently shown that their retrospective reports of childhood abuse were likely to underestimate, rather than overestimate, the actual occurrence of abuse (Femina et al., 1990; Williams, 1995). Another threat to validity is that the occurrence of an adverse event may not be the critical factor *per se*, but that one should also take into consideration the type of the event, as well as it’s frequency, intensity, and duration. Therefore, future studies may consider using more detailed and specific scales, such as the *Early Life Stress Inventory* (ref. in Opacka-Juffry and Mohiyeddini, 2012).

The use of parent reporting in assessment of child temperament has also been the subject of much controversy, particularly regarding construct validity due to subjective influences on the reporting process (Seifer and Sameroff, 1986; Kagan and Snidman, 2004; Rothbart, 2012). Some have taken the position that parent ratings have both objective and subjective components, and that the subjective components could reflect parents’ appraisals and feelings about their children’s behavior as much as constituting error variance (Bates and Bayles, 1984; Seifer, 2000). Nevertheless, the validity of the temperament dimensions used in the present study is strengthened by showing satisfactory fit in CFAs, even if it does not exclude the possibility of biases (Olafsen et al., 2018).

As to the association between infant temperament and parenting stress at 12 months, one should be cautious in interpreting the direction of effects. We do not know if infants’ temperamental characteristics influence parenting stress or, conversely, if parental stress influences how infant temperament is perceived.

Finally, in future studies, children’s attachment to their father and to their mother, as well as its association with parental attachment style and parenting stress, should also be examined.

## Conclusion

This study found that maternal attachment style assessed during pregnancy was related to parenting stress at 12 months after birth when a variety of potential risk factors were considered. A link between maternal ACEs and later parenting stress was also found, and maternal attachment style operated as a mediator in this link. In addition, it was demonstrated that perceived infant temperament contributed significantly to parenting stress 1 year after birth. The study illustrates the importance of understanding the multifactorial antecedents of parenting stress. Such knowledge may inform intervention efforts aimed at supporting mothers and their partners during the potentially difficult transition to parenthood. Study results emphasize the importance of working with prospective mothers and the attachment relationship with their partners already during pregnancy. Specifically, the role of maternal ACEs in determining later parenting stress, as well as the need to help mothers with a background of difficult relational experiences and an insecure attachment style before childbirth, should be acknowledged. After childbirth, promoting positive and sensitive parenting through attachment-based intervention, with a focus on infant temperament and behavioral individuality, may help mothers to better understand infant signals and behavior and thus reduce parenting stress.

## Ethics Statement

All procedures were in accordance with the ethical standards of the institutional and/or national research committee (Norwegian Regional Committees for Medical and Health Research Ethics, reference number 2011/560) and with the 1964 Helsinki Declaration and its later amendments or comparable ethical standards.

## Author Contributions

VM and LS planned and developed the Little in Norway study design. VM, TvS, EF, KO, and LS all contributed in planning of the design of the present paper. EF, TvS, and VM performed analysis of data. VM took the main responsibility for drafting the article, while TvS, EF, KO, and LS contributed substantially in revising it critically for important intellectual content. All authors approved the final draft for publication.

## Conflict of Interest Statement

The authors declare that the research was conducted in the absence of any commercial or financial relationships that could be construed as a potential conflict of interest.
